# Monitoring and discharging children being treated for severe acute malnutrition using mid-upper arm circumference: secondary data analysis from rural Gambia

**DOI:** 10.1093/inthealth/ihx022

**Published:** 2017-07-06

**Authors:** Alice Burrell, Marko Kerac, Helen Nabwera

**Affiliations:** a London School of Hygiene & Tropical Medicine, London WC1E 7HT, UK; b Leonard Cheshire Disability & Inclusive Development Centre, Department of Epidemiology & Child Health, University College London, London, UK; c The MRC Gambia Unit, Keneba, The Gambia

**Keywords:** Discharge, Mid-upper arm circumference, MUAC, SAM, Severe acute malnutrition, Wasting

## Abstract

**Background:**

Severe acute malnutrition (SAM) is a major public health problem. Mid-upper arm circumference (MUAC) is widely used to admit children to treatment programmes. However, insufficient data supporting MUAC discharge criterion limits its use as a stand-alone tool. Our aim was to evaluate MUAC for monitoring nutritional recovery and discharge.

**Methods:**

This was a secondary analysis of clinical data from children 6–59 months-old treated for SAM from January 2003 to December 2013 at the Nutritional Rehabilitation Unit in rural Gambia. Weight, weight-for-height z-score (WHZ) and MUAC response to treatment were assessed. Treatment indicators and regression models controlled for admission measurement and age were compared by discharge MUAC and WHZ.

**Results:**

Four hundred and sixty-three children with marasmus were included. MUAC, WHZ and weight showed parallel responses to treatment. MUAC≥125 mm as a discharge criterion performed well, showing good prediction of default and referral to hospital, acceptable duration of stay, and a higher absolute MUAC measure compared to WHZ≥−2.00, closely related to lower risk of mortality.

**Conclusions:**

MUAC can be used as a standalone tool for monitoring nutritional recovery. MUAC≥125 mm performs well as a discharge criterion; however, follow-up data is needed to assess its safety. Further research is needed on children meeting MUAC discharge criterion but with WHZ≤2.0.

## Introduction

Undernutrition accounts for just under half of all deaths in children aged under 5 years worldwide. Severe acute malnutrition (SAM) is a particularly important type of undernutrition responsible for over 500 000 deaths per year.^1,2^ Prevalence estimates suggest around 17 million children globally are currently suffering from SAM.^3^

What is today called SAM comprises two forms of malnutrition: wasting and/or kwashiorkor (oedematous malnutrition). Wasting was initially defined by a low weight-for-height z-score (WHZ);^4^ more recently an unadjusted mid-upper arm circumference (MUAC) has also been used as an independent criterion.^5^ Studies comparing the two measures found MUAC to be better than WHZ at predicting mortality, with deaths highest in those with a MUAC<115 mm.^6–9^ Also stimulating the rise of MUAC in nutrition programming was the shift, in the 2000s, from an inpatient-focused model of care to the ‘Community Management of Acute Malnutrition’ (CMAM). CMAM emphasised high programme coverage with outpatient treatment for clinically stable (uncomplicated) SAM cases. Early identification of affected children and active community case finding are key to CMAM's success.^10^ Towards these aims, unadjusted MUAC has many advantages over WHZ: it is cheap, simple, quick and acceptable^11^; colour-coded tapes mean that illiterate carers or fieldworkers can easily interpret measurements. A recent study found mothers can correctly use colour-coded MUAC tapes, increasing early detection.^12^ In contrast WHZ assessment requires: scales; a length/height board that can be troublesome to transport and use in field settings, especially with young infants; and sufficient numeracy and literacy to use a look-up table to convert raw measurements into a WHZ category.

MUAC was eventually endorsed by WHO and other UN agencies as an independent diagnostic criterion for SAM.^13,14^ The latest guidelines define SAM in 6–59 month-olds as: WHZ<−3.0 (with reference to the 2006 WHO growth standards) and/or MUAC<115 mm and/or bilateral pitting oedema.^15^ However, despite the practical advantages of MUAC and its widespread use in CMAM programmes, there have been many debates about whether it should, or could, replace WHZ entirely.^16–19^

Whilst the validity of MUAC-only based enrolment into nutritional care is well established,^8,9,19–21^ evidence for its use monitoring patient progress and deciding on readiness for discharge is limited to one recent study of outpatient treatment of SAM in Malawi. They presented evidence that MUAC≥125 mm as a discharge criterion was associated with low levels of relapse and mortality during a 3-month follow-up.^22^ The limited evidence base is a critical barrier to MUAC-only programming. Despite latest guidance suggesting using MUAC≥125 mm for discharge on the basis that mortality risk is very low above this threshold, more data are needed to know the implications of this recommendation.^11,15^

Our study sought to assess the adequacy of using MUAC for monitoring nutritional recovery, by confirming that changes in MUAC reflect response to treatment, and to assess the use of MUAC criteria for the discharge of children admitted with SAM. We used data from 10 years of admissions to a rural Nutrition Rehabilitation Unit (NRU) in The Gambia, a West African country with low HIV exposure. By evaluating the changes in MUAC, WHZ and weight during nutritional rehabilitation and at discharge, we aimed to evaluate MUAC and WHZ performance as tools for SAM treatment monitoring and discharge.

## Materials and methods

### Study setting

The Gambia is situated in West Africa and is home to less than 2 million people. Malnutrition is a significant public health problem in children under 5 years, with recent statistics reflecting serious levels of wasting (≥10%).^23^

The Medical Research Council (MRC), Keneba NRU is located in the West Kiang region with a population of 15 117.^24^ It is integrated into a clinic that has been providing free primary health care services for over four decades. The NRU currently admits some 70–100 children per year and provides outpatient care following limited inpatient care (maximum of 48 hours) using Therapeutic milk (F75, F100) Ready-to-Use-Therapeutic Food (Plumpynut^®^; Malaunay, France)^25^ and/or enriched pap (maize meal porridge with milk, oil and ground nut paste). Historically, discharge from the MRC Keneba NRU outpatient phase was at WHZ≥−2.00 (National Centre for Health Statistics [NCHS] growth references until 2006) but with the roll-out of CMAM in 2013, children with a WHZ<−3.0 (WHO growth standards) and no medical complications are now transferred to community-based care for continued care.

### Study design and study population

This was an observational, retrospective secondary data analysis of routinely collected anthropometric data from children aged 6–59 months-of-age who were admitted for the first time to the MRC Keneba NRU with a diagnosis of: ‘marasmus’; ‘severe acute malnutrition’ or ‘protein energy malnutrition’ between January 2003 and December 2013. We included those with severe wasting (marasmus) defined as WHZ<−3.0 (WHO growth standards) and/or MUAC<115 mm. From 2008–2009 the NRU used a MUAC cut-off of 110 mm, prior to this MUAC was not used for admission but only WHZ<−3.0 and/or oedema. We excluded children with kwashiorkor from our analysis due to the different weight gain trends over treatment and the small numbers in the population. We also excluded any readmissions since these were more likely to be atypical cases with complex problems underlying SAM.

Height, weight and MUAC were recorded at admission, weekly and at discharge by NRU staff. Height was recorded to the nearest millimetre using a Raven Kiddimetre (Raven Equipment, Great Dunmow, Essex, UK); weight was recorded to the nearest 10 grams using electronic Seca 336 sitting scales (Chasmors Ltd, London, UK); MUAC was measured using various MUAC tapes, differing over time and including both colour coded and plain tapes; however, all measured to the nearest millimetre.

Data for this secondary analysis was extracted from the MRC Keneba NRU database. This comprised patient data entered in Access (Microsoft Corp., Redmond, WA, USA) soon after a child was discharged, using a double-entry method for validation. Oedema status had not been captured initially so was sourced from hard copy patient files in July 2014.

Z-scores were calculated using Emergency Nutrition Assessment (ENA) for SMART Software (Version October 2007)^26^ with reference to the 2006 WHO Multicentre Growth Reference Study (MGRS) growth curves using weight and height as recorded in the database, and age calculated by ‘date of admission–date of birth’.^27^

Recovery was defined as MUAC≥125 mm at discharge, as recorded in the database, and/or WHZ≥−2.00 at discharge as calculated from weight and height recorded in the database, with reference to WHO MGRS growth curves. The following recovery variables were defined as follows: recovered by MUAC (%): (number discharged at MUAC≥125 mm/total number of children)*100; not recovered by MUAC (%): (number discharged at MUAC<125 mm/total number of children)*100; recovered by WHZ (%): (number discharged at WHZ≥−2.00/total number of children)*100; and, not recovered by WHZ (%): (number discharged at WHZ<−2.00/total number of children)*100.

Recovery indicators were calculated as: Weight gain=([discharge weight (g)−minimum weight (g)]/minimum weight (kg))/length of stay (days); MUAC gain=(discharge MUAC (mm)− minimum MUAC (mm))/length of stay (days); Length of stay= number of days treated at the NRU (day of admission=1).

Treatment outcomes were assessed by: readmissions (%): (number of first readmissions to the NRU within study timeframe/number of first admissions within the study timeframe)*100; default: carer leaving against medical advice or absconding; death: death whilst in treatment at the NRU (follow up information on short-term mortality not available); and referral: referral to hospital or health centre when patients had medical complications which were beyond the capability of the NRU to treat.

### Statistical methods

We used STATA software version 13.1(StataCorp LP, College Station, TX, USA) for all statistical analysis.^28^ Distributions were first visually assessed for normality. Outliers were identified and the standard cleaning criteria applied to admission anthropometric measurements, on the basis that they more likely represent data errors than true values;^29^ cases meeting the following criteria were excluded from analysis: −6.00>WHZ>−1.00; height-for-age z-score (HAZ)<−6.00; WAZ<−6.00; 80 mm<MUAC<140 mm; weight gain>30 g/kg/day.

Appropriate average measures were calculated for analysis. Means (SD) were reported for variables with approximate normal distribution (weight, height, WHZ, HAZ, MUAC, weight gain) and medians (IQR) or geometrical means (IQR) for those with skewed distributions (age, length of stay). Data trends were explored graphically for descriptive analysis.

For comparison of treatment outcomes and indicators of WHZ to MUAC discharge thresholds, χ^2^ tests were used for comparisons across categorical outcomes, and t-tests were used for comparisons of quantitative variables across binary outcomes. For comparison of treatment outcomes and indicators across all discharge categories (met WHZ criteria only, met MUAC criteria only, met both criteria, met none of the criteria) ANOVA and Scheffe's test were used when the outcome was categorical and non-parametric trend tests were used to test for linear trends of a non-normal quantitative variable across categories.

Pearson's correlation coefficient was used to test for correlations between weight, WHZ and MUAC as well as between MUAC gain and weight gain. Logistic regression was used to test the linear relationship of treatment indicators (weight gain and length of stay) with binary discharge categories of MUAC≥125 mm and WHZ≥−2.0. Potential confounders and modifiers such as age at admission, sex and presence of stunting (HAZ<−2.0) were tested in models and respective admission measurements were controlled for. Treatment length was log transformed for comparisons between groups by t-test and for use in logistical regression as a quantitative variable. ROC-curves were generated using Stata Software to test how weight gain over treatment predicted discharge above dichotomised discharge threshold variables, set as: MUAC≥125 mm and WHZ≥2.0.

Cases with random missing measurements were excluded. Cases with non-random missing data were made in to a sub-group and compared to cases with complete data for admission measurements before exclusion.

## Results

### Participants

**Figure 1.**
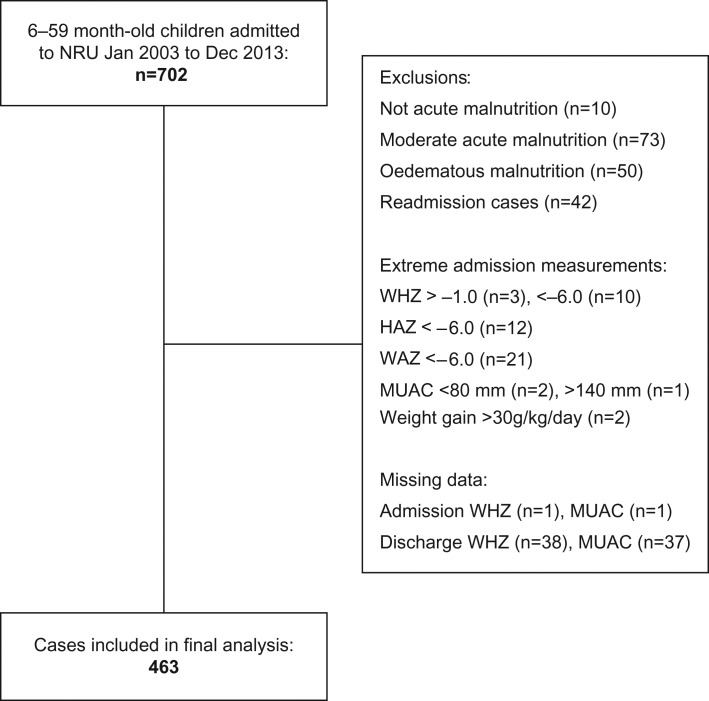
Flow chart showing participant selection with exclusions on case criteria, extreme values and missing data. Criteria are not mutually exclusive. Overall 239 cases were excluded, leaving 463 eligible cases in the study population.

The flow chart in Figure 1 outlines how eligible children were selected, summarising exclusions on case criteria, extreme values and missing measurements.

Four hundred and sixty-three children with marasmus were included in the final analysis. The majority of cases (455/465; 97.8%) were under 36 months-old. High levels of severe underweight (394/465; 84.7%) and stunting (267/465; 57.4%) underlying the marasmus were evident (Table 1). There was a 59.8% (279/465) overlap between WHZ and MUAC in SAM admission criteria in this population, 32.8% (152/465) met only WHZ admission criteria, and 6.9% (32/465) met only MUAC admission criteria (Supplementary Figure 1).

**Table 1.** Demographics of the 463 children with marasmus included in the study

Baseline data	n/mean	%/SD
Median age, months (IQR)	14	11, 20
6–11 months	159	34.3%
12–23 months	243	52.5%
24–35 months	53	11.5%
36–47 months	7	1.5%
48–59 months	3	0.7%
Male	256	55.3%
Mean weight, kg (SD)	6.36	1.13
Severely underweight (WAZ<−3.0)	394	84.7%
Mean height, cm (SD)	72.0	6.5
Stunted (HAZ<−2.0)	267	57.4%
Mean WHZ (SD)	−3.78	0.66
Mean MUAC, mm (SD)	111	93

HAZ: height-for-age z-score; MUAC: mid-upper arm circumference; WAZ: weight-for-age z-score; WHZ: weight-for-age z-score.

### Outcome data

On average, absolute weight gain was 1.04 kg (SD=0.60), absolute MUAC gain was 11 mm (IQR=6; 16) and absolute WHZ gain was 1.68 (SD=0.88). The mean rate of weight gain was 8.9 g/kg/day (SD=4.7) and mean rate of MUAC gain was 0.6 mm/day (IQR=0.3; 0.8). The median time in treatment was 18 days (IQR=13; 25).

By MUAC, 36.1% (167/463) of children admitted were classified as recovered, whilst 63.9% (296/463) did not recover. By WHZ, 45.6% (211/463) recovered, whilst 54.5% (252/463) did not. Other treatment outcomes included: 4.1% (n=19) defaulted, 3.2% (n=15) were referred to hospital and 1.1% (n=5) died whilst in treatment. Over the 10 years, 6.0% (n=32) of patients were readmitted.

### Monitoring nutritional recovery

**Figure 2.**
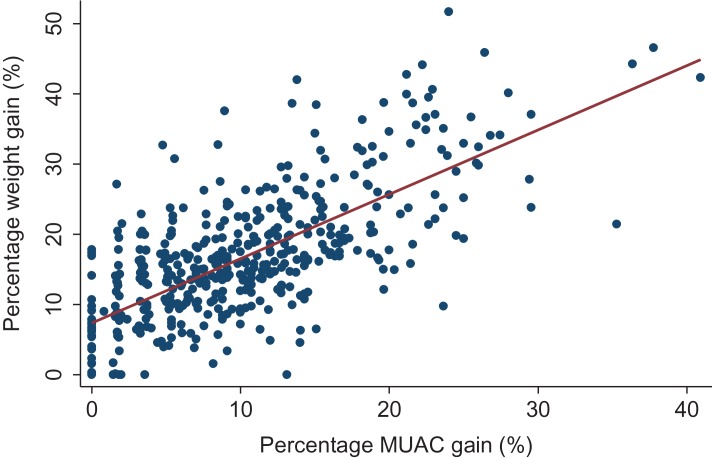
The positive linear correlation between percentage weight gain and percentage MUAC gain, for all 463 marasmus cases included in the study population. Dots (percentage weight gain and percentage MUAC gain); Line (fitted line to demonstrate positive correlation).

Average weight, WHZ and MUAC ran in parallel, both increasing during treatment. There was significant positive correlation between percentage MUAC gain (mm) and percentage weight gain (g) (r^2^=0.50, r=0.71; p<0.001) (Figure 2).

The mean percentage MUAC gain was 10.2% (SD=7.4). Weight gain showed similar patterns with an average of 16.8% (SD=9.7). There were a number of cases with 0% MUAC gain; when explored the majority of these were cases referred to hospital (Supplementary Figure 2).

### Discharge criteria

Table 2 summarises treatment indicators and outcomes in relation to WHZ and MUAC discharge criteria (see Supplementary Table 1 for full results by thresholds). Those ending their stay with WHZ≥−2.0 had a statistically higher mean rate of weight gain than those ending treatment with MUAC≥125 mm (diff: −1.44 [−2.34, −0.54]; p=0.001) and a higher average WHZ gain (diff: −0.014 [−0.03, −0.002]; p=0.02). Those ending their stay with MUAC≥125 mm had a statistically higher MUAC on discharge than those with WHZ≥−2.0 (diff: 60.0 [46.0, 74.0]; p<0.001). There were no significant differences in: mean MUAC gain (p=0.47), length of stay (p=0.85) or number of readmissions (p=0.17). The number referred to hospital was zero for both and defaulters were too small a number to draw any valid conclusions.

**Table 2.** Average treatment and discharge outcomes for children who reached either MUAC and/or WHZ discharge criteria. Results of statistical significance tests are shown, comparing outcomes between discharge by MUAC and discharge by WHZ

Mean treatment indicators and outcomes (SD)	MUAC≥125 mm (n=167)	WHZ≥−2.0 (n=211)	Difference [95% CI]	p-value
Weight gain (g/kg/day)	9.6 (5.0)	11.0 (4.5)	−1.4 [−2.34, −0.54]	0.001
MUAC gain (mm/day)	0.74 (0.45)	0.71 (0.42)	0.03 [−0.06, 0.12]	NS
WHZ gain (z-score/day)	0.11 (0.06)	0.12 (0.06)	0.01 [0.03, −0.002]	0.023
Length of stay, days				
Median [IQR]	19 [13, 26]	18 [13, 26]		NS
Mean^a^ [95% CI]	18.6 [17.2, 20.1]	18.3 [17.1, 19.6]	NA	NA
Discharge MUAC, mm	131 (48)	125 (80)	60 [46, 74]	<0.001
Discharge WHZ	−1.82 (0.72)	−1.53 (0.38)	−0.29 [−0.40, −0.18]	<0.001
No. defaulted	3 (1.8%)	2 (0.9%)	NA	NA
No. referred to hospital	0	0	NA	NA
No. readmission	12 (7.1%)	8 (3.8%)		NS

MUAC: mid-upper arm circumference; NA: not applicable (sample size too small); NS: not significant, p≥0.05; WHZ: weight-for-height z-score.

^a^ geometric mean.

Table 3 shows that overall those with MUAC≥125 mm at end of stay had good outcomes and treatment indicators. However, cases who only met MUAC discharge criteria (i.e., WHZ<−2.0) by end of stay had lowest weight gain on average at 7.0 g/kg/day (SD=4.0), significantly lower than those who met only WHZ discharge criteria (Scheffe's test; p<0.001) and those who met both discharge criteria (Scheffe's test; p<0.001). It did not differ significantly from those who did not meet either discharge criteria (Scheffe's test; p=0.72). Table 3 also shows in comparison to those meeting both discharge criteria, those meeting MUAC discharge only had a significantly lower MUAC gain (p=0.0016), but no difference in length of stay (p=0.88) or readmissions (p=0.11). Number of defaulters was too small to draw any valid conclusions.

**Table 3.** A comparison of outcomes by WHZ status for cases meeting MUAC discharge (MUAC≥125 mm)

Treatment indicators and outcome	Overall (n=168)	WHZ≥−2 (n=113)	WHZ<−2 (n=54)	Diff.	95% CI	p-value
Mean weight gain (g/kg/day) (SD)	9.6 (5.0)	10.8 (5.0)	7.0 (4.0)	3.58	2.01, 5.14	<0.001
Mean^a^ length of stay, days	19	18.4	18.7			NS
[95% CI]	[13, 26]	[16.7, 20.4]	[16.3, 21.3]			
Mean MUAC gain (mm/day) (SD)	0.7 (0.4)	0.8 (0.4)	0.6 (04)	−0.23	−0.38, −0.09	0.0016^a^
No. of defaulters	3 (1.8%)	0 (0%)	3 (5.6%)	NA	NA	NA
No. of readmissions	12 (6%)	5 (4%)	7 (13%)			NS

MUAC: mid-upper arm circumference; NA: not applicable (sample size too small); NS: not significant, p≥0.05; WHZ: weight-for-height z-score.

^a^ geometric mean (significance test not possible with geometric means).

Those who ended their stay with MUAC<125 mm were significantly younger (diff: 4.07 [2.73, 5.41]; p<0.001), more likely to be female (p=0.0060) and more likely to be stunted (p=0.001) at admission than those ending their stay with MUAC≥125 mm.

### Logistic regression models

Logistic regression applied to the outcome of MUAC≥125 mm, adjusting for respective admission measurement and age, confirmed that length of stay (days) was not significantly associated with a case being at MUAC≥125 mm (OR: 1.25 [0.88, 1.77]; p=0.22) or at WHZ≥−2.0 (OR: 1.19 [0.85, 1.67]; p=0.31) at end of stay (see Supplementary File 1 for regression models).

Logistic regression showed that ending stay with MUAC≥125 mm (OR: 1.06 [1.00, 1.11]; p=0.032) and WHZ≥−2.0 (OR: 1.27 [1.20, 1.35]; p<0.001) predicted higher weight gain (g/kg/day). The MUAC≥125 mm model explained 26% of the variation, more than that of the equivalent WHZ≥−2.0 model at 20%. Supplementary Figure 3 shows this graphically, with a greater area under the curve (AUC) in the MUAC ROC curve.

### Other analyses

Sensitivity analyses were run for cases with non-random missing data (Supplementary File 2). Neither including cases with missing discharge MUAC (n=8) or WHZ (n=7) only, or excluding those missing oedema status (n=12) made a significant difference to the overall results. Controlling for area of inhabitance also made no significant difference to the overall results. Defaulters (n=19) had a significantly lower admission MUAC (diff: 0.46 [95% CI 0.038, 0.891] p=0.033) than non-defaulters. There was no significant difference in any other admission measurements.

## Discussion

### Monitoring nutritional recovery

Our results support the hypothesis that serial MUAC measurements are suitable for monitoring nutritional recovery: over the days of treatment observed, there was a clearly observable increase in both absolute MUAC and percentage MUAC change from baseline. Another key finding was that MUAC changes ran in parallel to WHZ and weight, with percentage MUAC change showing a positive correlation with percentage weight change. A recent paper demonstrated this correlation between MUAC change and weight change in data from three separate countries: Ethiopia, Malawi and Bangladesh. They also found that MUAC and weight showed similar changes during periods of illness during SAM treatment, both reducing rapidly.^30^

These findings are consistent with reports from a large scale analysis of 24 792 patients of a therapeutic feeding programme in Burkina Faso which used MUAC for admission.^31^ One programme in India also reported MUAC to function as an acceptable monitoring tool after assessing discharge WHZ, length of stay and average weight gain of cases admitted and discharged on MUAC.^32^ Médecins Sans Frontières (MSF) have since adopted MUAC-only programming for SAM treatment programmes, with successful programme outcomes.^19^

### Discharge criteria

Our results showed that discharge above both MUAC and WHZ thresholds of ≥125 mm and ≥−2.0, respectively, predicted a higher rate of weight gain. However, MUAC had a greater predictive ability for weight gain when controlled for admission age and admission measurement. Length of stay was not found to differ by MUAC or WHZ status at discharge. MSF reported similar findings from a CMAM programme in North Sudan using MUAC ≥125 mm for discharge. When 753 cured cases were reclassified by their WHZ status similar trends were seen in weight gain and length of stay.^33^ When interpreting weight gain with relation to MUAC we must keep in mind that weight gain is not a ‘gold-standard’ for recovery rate.

Our results showed that, despite lower weight gain, those ending their stay with MUAC≥125 mm had on average a 6 mm higher MUAC than those ending their stay with WHZ ≥−2.0. Absolute MUAC has been shown to be closely related with mortality, more so than low weight gain, which implies that those discharged at the MUAC threshold may be less at risk than those discharged at the WHZ threshold.^11^

Perhaps more importantly defaulters and length of stay did not differ significantly by MUAC or WHZ status at discharge. There is indication that readmissions do not differ, however as there was no standard follow-up process after discharge, data may not be fully representative. Both a low MUAC and low WHZ predicted default and referral to hospital as well as all inpatient deaths being below both thresholds. In fact in those referred we see very little MUAC gain; as these cases would have been referred early on during the intensive phase MUAC and weight gain would not have begun.

### MUAC-only programming

MUAC predicts outcomes and treatment progress similar to WHZ in this population. One potential implication of this observation is that programme outcomes would be similar if MUAC alone was used for discharge. This would enhance existing arguments for MUAC-programming: simplicity and coverage; and better reliability and validity than WHZ measurements.^11,21,34^ A concern with replacing WHZ entirely is the difference in populations identified as SAM: this is different in different populations.^35^ Studies in South-East Asia show MUAC to identify a much smaller population of SAM children than WHZ;^36,37^ whereas in Kenya a 70% overlap has been reported.^8^ It has been suggested that these geographical differences are due to differences in body shape.^20^ MUAC also identifies a younger, more female and more stunted population ‘at-risk’ which some argue is a desirable characteristic,^8,18^ whilst others argue that the use of WHZ should continue as it effectively controls for these factors.^17^

In our population only 24% fulfil both discharge criteria simultaneously. Those meeting MUAC discharge only criteria in our population i.e., MUAC≥125 mm with WHZ<−2.0 had significantly lower weight gain and MUAC gain than those meeting both MUAC and WHZ criteria. Importantly from a safety viewpoint, there was indication in our data that this was not associated with an increase in readmissions. This finding is supported by a recent study which included follow-up data on 258 children treated for SAM in Malawi. Children were discharged by MUAC≥125 mm and followed up for 3 months. They observed low levels of relapse to SAM and low mortality, concluding that MUAC was a safe discharge criterion.^22^

However, an MSF therapeutic feeding programme in Bihar reported a significantly higher relapse rate in a minority of cases (2%) who were discharged by MUAC but with WHZ<−3.0.^38^ The question which must be answered is whether this minority are ‘at-risk’ of mortality. A study in Bangladesh showed that children at WHZ<−3.0 did not deteriorate over 3 months when left without nutritional intervention and in fact some improved, but this evidence base needs strengthening and careful follow-up of cases discharged by MUAC-only is recommended.^39^

### Limitations

The limitations of this study include that the Keneba Electronic Medical Records System (KEMReS) did not alert for low MUAC during the time of the study, but only for low WHZ. MUAC screening was also not commonplace in this area and hence most admissions were based on WHZ. Only 7% were admitted on MUAC only meaning the study sample is not fully representative of the SAM population and results may not reflect the true results, limiting extrapolation. In addition stunting is at a serious level in this population, meaning results may not apply so well to less stunted populations. We were also unable to access adequate admission morbidity data to include in this analysis.

Our data is from a treatment programme which delivers care through initial (maximum 48 hours) inpatient treatment, therefore findings may not be generalisable to the outpatient treatment approach (CMAM) currently used in the majority of contexts.

Community programmes varied in extent and were limited, this means that many relapses may have been identified through passive referral. In addition, the follow-up procedures in place relied on patients re-presenting to the clinic for review at 1 week and 4 weeks after discharge with no community follow-up. This likely underestimates the number of patients who were readmitted and means outcomes of the majority of children after treatment are unknown. However, this is useful baseline information for this population, demonstrating previous findings in a novel context. Future work should incorporate the follow-up of these patients.

The use of operational data limits the study; Anthropometry for instance was only measured once rather than double measured as for research studies. However, large numbers mean that the resulting ‘noise’ in the data does not affect our final conclusions so much. Missing data is also a limitation. Missing discharge data was mainly due to default. Despite defaulters having significantly lower admission MUAC and weight gain, a sensitivity analysis excluding missing data cases made no significant difference to our overall results. Missing data and defaulters are a reality in any nutritional programme; since numbers were low in ours, we do not believe this made any major differences to our conclusions.

The fact that SAM case definitions have changed over the 10-year study period may limit the generalisability of our results to current SAM treatment programmes. Reference data for WHZ changed from NCHS to MGRS WHO growth curves in 2006, meaning calculated WHZ values for analysis will not be those used at the time for cases before 2006. However, running analyses using WHZ with reference to NCHS data made little difference to results. On the positive side, at least protocols for treatment have been stable over the period of our investigation.

### Conclusions

We conclude that monitoring MUAC changes has potential for monitoring treatment progress and nutritional recovery of children in treatment for SAM: MUAC tracks well alongside weight and WHZ changes. MUAC ≥125 mm shows potential as a discharge criterion, predicting treatment outcomes with a similar ability to WHZ and leading to the same average length of stay. Rate of weight gain is lower in those meeting MUAC ≥125 mm at discharge but there do not appear to be adverse consequences of this in terms of there being no significant difference in numbers of readmissions. Additionally, those meeting the MUAC threshold at end of stay showed a higher absolute measure of MUAC which has been shown to be related to a lower risk of mortality, than those meeting the WHZ threshold. Future research could focus on longer term outcomes of these cases and further refine the criteria for monitoring the rate of recovery and discharge. In the meantime, if MUAC-only programming is used, careful follow-up is advised particularly for those children whose MUAC gain is slow. We encourage routine reporting of MUAC gain over treatment to enable comparisons between nutritional programmes and setting of ideal rates, as is currently in place for weight gain.

## Supplementary data

Supplementary data are available at *International Health* online (http://inthealth.oxfordjournals.org/).

## Supplementary Material

Supplementary Data

Supplementary Data

Supplementary Data

Supplementary Data

Supplementary Data

Supplementary Data
