# Supplementary material for: Monitoring and discharging children being treated for severe acute malnutrition using mid-upper arm circumference: secondary data analysis from rural Gambia
Source: Int Health. 2017 Jul 6;9(4):226–33. doi: 10.1093/inthealth/ihx022 (PMC5881269; doi:10.1093/inthealth/ihx022)
Supplement: Supplementary Data [file edsupplementaryfigure2.docx]

Supplementary Figure 2. Histogram showing overall percentage MUAC gain over treatment for 463 marasmus cases

MUAC: mid-upper arm circumference
