# Supplementary material for: Monitoring and discharging children being treated for severe acute malnutrition using mid-upper arm circumference: secondary data analysis from rural Gambia
Source: Int Health. 2017 Jul 6;9(4):226–33. doi: 10.1093/inthealth/ihx022 (PMC5881269; doi:10.1093/inthealth/ihx022)
Supplement: Supplementary Data [file edsupplementaryfile1.docx]

Supplementary File 1. Logistic regression models

A. Logistic regression models of MUAC ≥125 mm at discharge by weight gain, admission age, admission MUAC and sex

|  | MUAC ≥125 mm | Odds ratio | 95% CI | p-value | Pseudo R^2^ |
| --- | --- | --- | --- | --- | --- |
| Model 1 | Weight gain (g/kg/day) | 1.04 | 1.00, 1.09 | 0.037 | 0.006 |
| Model 2 | Weight gain (g/kg/day) | 1.06^a^ | 1.00, 1.11 | 0.032 | 0.059 |
|  | Admission age (months) | 1.08^a^ | 1.05, 1.11 | <0.001 |  |
| Model 3 | Weight gain (g/kg/day) | 1.05^a^ | 1.00, 1.11 | 0.048 | 0.268 |
|  | Admission MUAC (cm) | 4.34^a^ | 3.02, 6.23 | <0.001 |  |
|  | Admission age (months) | 1.08^a^ | 1.04, 1.12 | <0.001 |  |
|  | Stunting (Yes-No) | 0.61^a^ | 0.37, 1.02 | NS |  |
|  | Sex (Female-Male) | 0.71^a^ | 0.44, 1.13 | NS |  |
| Model 4 | Weight gain (g/kg/day) | 1.06^a^ | 1.00, 1.11 | 0.032 | 0.260 |
|  | Admission MUAC (cm) | 4.93^a^ | 3.48, 6.98 | <0.001 |  |
|  | Admission age (months) | 1.07^a^ | 1.03, 1.11 | <0.001 |  |

B. Logistic regression models of WHZ ≥ -2.0 at discharge by weight gain, admission age, admission WHZ and sex

|  | WHZ ≥ -2 | Odds ratio | 95% CI | p-value | Pseudo R^2^ |
| --- | --- | --- | --- | --- | --- |
| Model 1 | Weight gain (g/kg/day) | 1.22 | 1.16, 1.29 | 0.001 | 0.119 |
| Model 2 | Weight gain (g/kg/day) | 1.27^a^ | 1.20 1.35 | <0.001 | 0.202 |
|  | Admission WHZ | 3.17^a^ | 2.21, 4.55 | <0.001 |  |
|  | Admission age (months) | 1.05^a^ | 1.01, 1.08 | <0.004 |  |
| Model 3 | Weight gain (g/kg/day) | 1.27^a^ | 1.20, 1.35 | <0.001 | 0.203 |
|  | Admission WHZ | 3.10^a^ | 2.14, 4.49 | <0.001 |  |
|  | Admission age (months) | 1.05^a^ | 1.01, 1.08 | <0.004 |  |
|  | Sex (Female-Male) | 1.13^a^ | 0.73, 1.75 | NS |  |

MUAC: mid-upper arm circumference; NS: not significant, p≥0.05; WHZ: weight-for-height z-score.

^a^ Odds ratio is adjusted for all other variable in the section of the table.
