# Supplementary material for: Monitoring and discharging children being treated for severe acute malnutrition using mid-upper arm circumference: secondary data analysis from rural Gambia
Source: Int Health. 2017 Jul 6;9(4):226–33. doi: 10.1093/inthealth/ihx022 (PMC5881269; doi:10.1093/inthealth/ihx022)
Supplement: Supplementary Data [file edsupplementaryfile2.docx]

Supplementary File 2. Sensitivity analyses

A. Significance tests: cases with complete data and those with non-random missing discharge data

|  | Mean (SD) | |  |
| --- | --- | --- | --- |
|  | Complete data (n=473) | Missing data (n=52) | p-value |
| Admission age (months) | 15.9 (7.7) | 14.9 (6.5) | NS |
| Male (%) | 55 | 60 | NS |
| Weight (kg) | 6.3 (1.2) | 5.9 (1.3) | 0.028 |
| Height (cm) | 71.7 (6.8) | 70.1 (7.3) | NS |
| WHZ | -3.8 (0.8) | -4.1 (1.1) | NS |
| MUAC (mm) | 11.1 (1.1) | 10.6 (1.1) | 0.0030 |
| Defaulters | 20 (4.2%) | 7 (14.6%) | <0.001^a^ |
| Referred to hospital | 16 (3.4%) | 20 (41.7%) |  |
| Cure | 210 (44.4%) | 13 (27.1%) |  |
| No cure | 227 (48.0%) | 8 (16.7%) |  |

MUAC: mid-upper arm circumference; NS: not significant, p≥0.05; WHZ: weight-for-height z-score.

^a^ χ^2^ test for association

B. Regression analysis: cases with missing WHZ and MUAC data cases

| MUAC ≥125 mm | Odds ratio | 95% CI | p-value | Pseudo R^2^ |
| --- | --- | --- | --- | --- |
| Weight gain (g/kg/day) | 1.04 | 1.00, 1.09 | NS | 0.0060 |
| Weight gain (g/kg/day) | 1.03^a^ | 0.99 1.07 | NS | 0.060 |
| Admission age (months) | 1.08^a^ | 1.05, 1.12 | <0.001 |  |
| Weight gain (g/kg/day) | 1.05^a^ | 1.00, 1.11 | NS | 0.27 |
| Admission MUAC (cm) | 4.32^a^ | 3.01, 6.19 | <0.001 |  |
| Admission age (months) | 1.08^a^ | 1.04, 1.12 | <0.001 |  |
| Stunting (yes-no) | 0.60^a^ | 0.36, 0.99 | 0.047 |  |
| Sex (female-male) | 0.69^a^ | 0.43, 1.11 | NS |  |
| Weight gain (g/kg/day) | 1.05^a^ | 1.00, 1.11 | 0.046 | 0.27 |
| Admission MUAC (cm) | 4.55^a^ | 3.18, 6.50 | <0.001 |  |
| Admission age (months) | 1.08^a^ | 1.04, 1.12 | <0.001 |  |
| Stunting (yes-no) | 0.63 | 0.38, 1.04 | NS |  |

MUAC: mid-upper arm circumference; NS: not significant, p≥0.05; WHZ: weight-for-height z-score.

^a^ Odds ratio is adjusted for all other variables in the section of the table.
