# Supplementary material for: Monitoring and discharging children being treated for severe acute malnutrition using mid-upper arm circumference: secondary data analysis from rural Gambia
Source: Int Health. 2017 Jul 6;9(4):226–33. doi: 10.1093/inthealth/ihx022 (PMC5881269; doi:10.1093/inthealth/ihx022)
Supplement: Supplementary Data [file edsupplementarytable1.docx]

Supplementary Table 1. Average treatment and discharge outcomes by MUAC and WHZ discharge thresholds

| Mean treatment indicators and outcomes (SD) | MUAC ≥125 (n=167) | WHZ ≥ -2.0  (n=211) | MUAC <125 (n=296) | WHZ < -2.0 (n=252) |
| --- | --- | --- | --- | --- |
| Weight gain (g/kg/day) | 9.6 (5.0) | 11.0 (4.5) | 8.6 (4.6) | 7.3 (4.3) |
| MUAC gain (mm/day) | 0.74 (0.45) | 0.71 (0.42) | 0.49 (0.36) | 0.47 (0.38) |
| WHZ gain (z-score/day) | 0.11 (0.06) | 0.12 (0.06) | 0.09 (0.05) | 0.07 (0.05) |
| Length of stay (days) |  |  |  |  |
| Median [IQR] | 19 [13, 26] | 18 [13, 26] | 18 [12, 25] | 18 [12, 25] |
| Geometric mean [95% CI] | 18.6 [17.2, 20.1] | 18.3 [17.1, 19.6] | NA | NA |
| Discharge MUAC (mm) | 131 (48) | 125 (80) | 116 (70) | 118 (90) |
| Discharge WHZ | -1.82 (0.72) | -1.53 (0.38) | -2.44 (0.84) | -2.79 (0.71) |
| No. defaulted | 3 (1.8%) | 2 (0.9%) | 16 (5.4%) | 17 (6.7%) |
| No. referred to hospital | 0 (0%) | 0 (0%) | 15 (5.1%) | 15 (6.0%) |
| No. readmission | 12 (7.1%) | 8 (3.8%) | 20 (6.7%) | 24 (9.5%) |

MUAC: mid-upper arm circumference; NA: not applicable; WHZ: weight-for-height z-score.
