# Supplementary material for: Monitoring and discharging children being treated for severe acute malnutrition using mid-upper arm circumference: secondary data analysis from rural Gambia
Source: Int Health. 2017 Jul 6;9(4):226–33. doi: 10.1093/inthealth/ihx022 (PMC5881269; doi:10.1093/inthealth/ihx022)

Supplementary Figure S1: Venn diagram of SAM admission criteria: WHZ < -3.0 and MUAC < 115mm for all 463 marasmus cases included.

MUAC: mid-upper arm circumference, WHZ: weight-for-age z-score

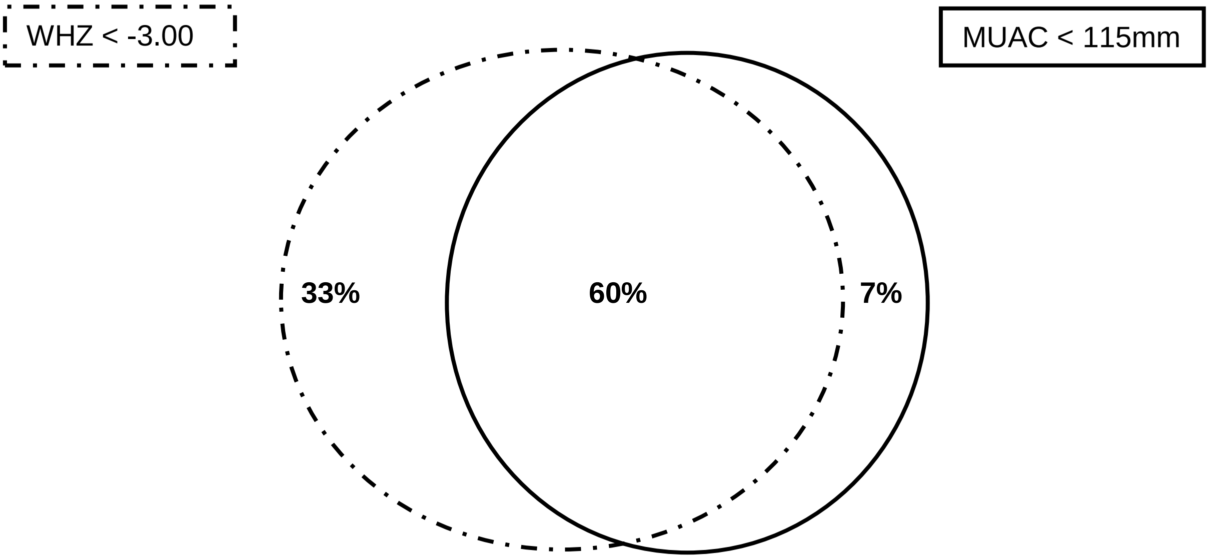

Supplement: Supplementary Data [file supplementaryfigures1.pdf]
