# Supplementary material for: Monitoring and discharging children being treated for severe acute malnutrition using mid-upper arm circumference: secondary data analysis from rural Gambia
Source: Int Health. 2017 Jul 6;9(4):226–33. doi: 10.1093/inthealth/ihx022 (PMC5881269; doi:10.1093/inthealth/ihx022)
Supplement: Supplementary Data [file supplementaryfigures3.pdf]

Figure S3: ROC curves for logistical regression models on WHZ ≥-2.0 and MUAC ≥125mm with: A. weight gain, B. weight gain adjusted for admission measurement and age and C. length of stay

A. **i. WHZ and ii. MUAC with weight gain**

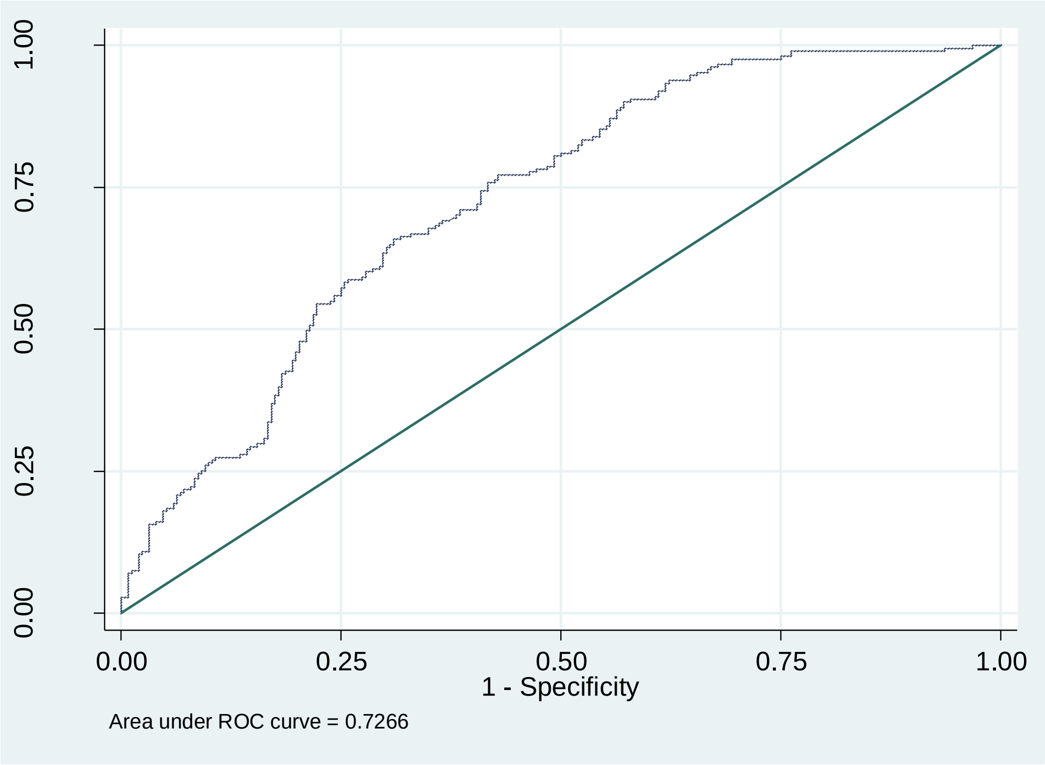

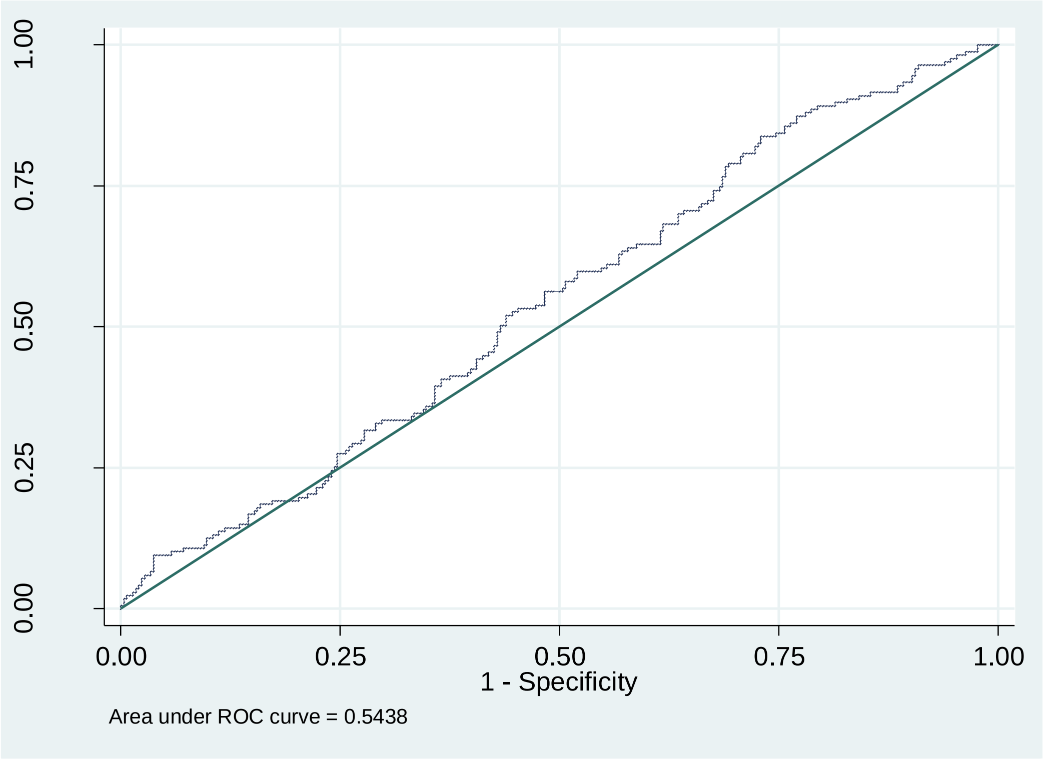

B. i. WHZ and ii. MUAC with weight gain controlled for admission measurement and age

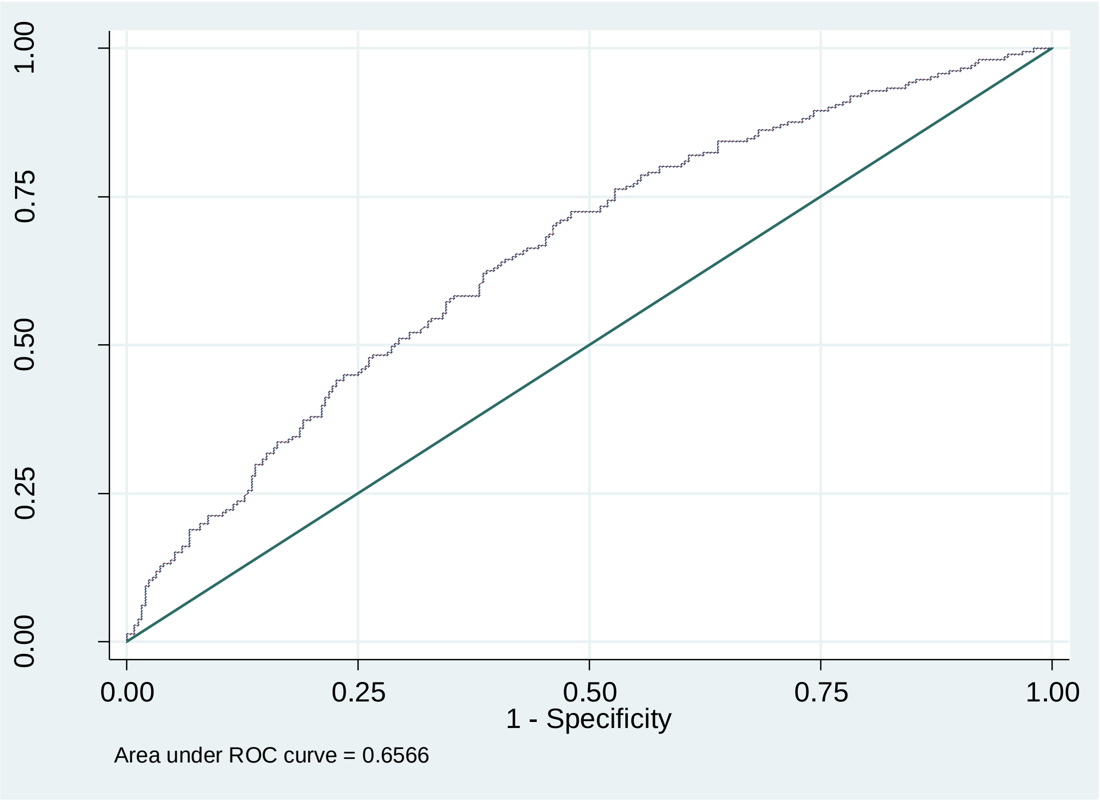

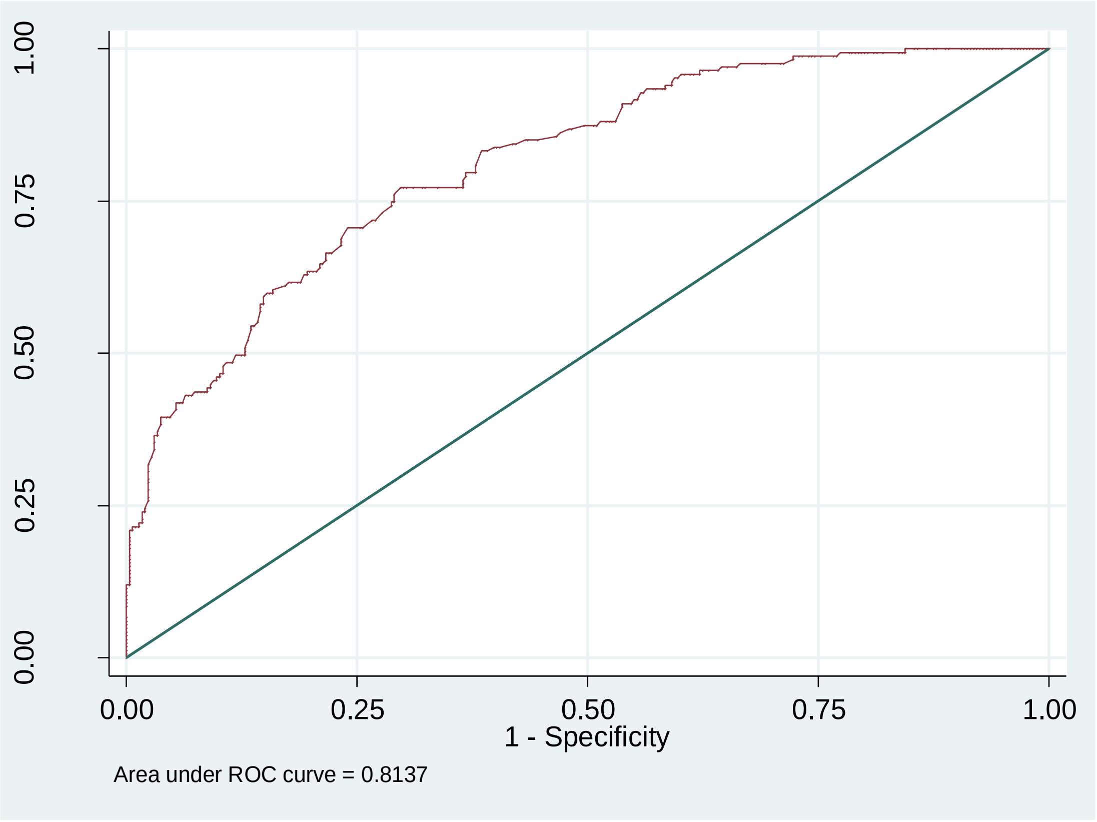

C. i. WHZ and ii. MUAC with length of stay

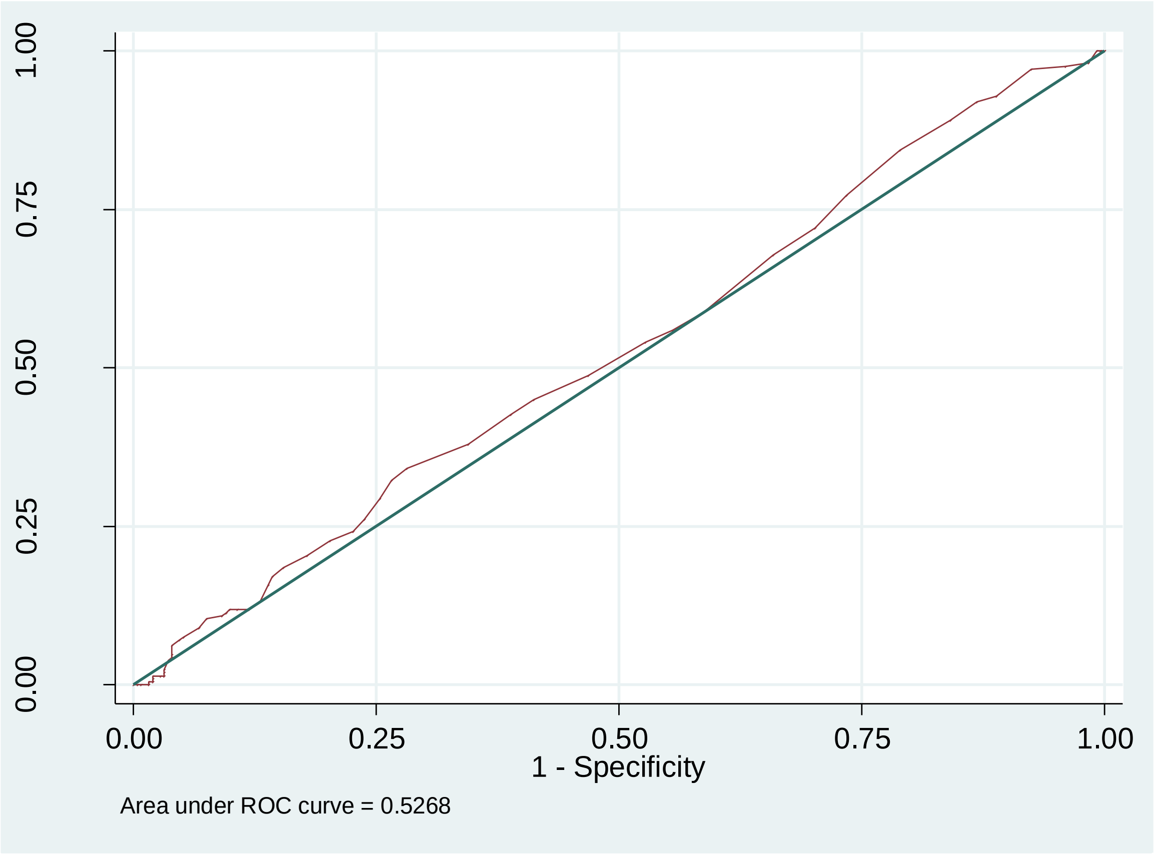

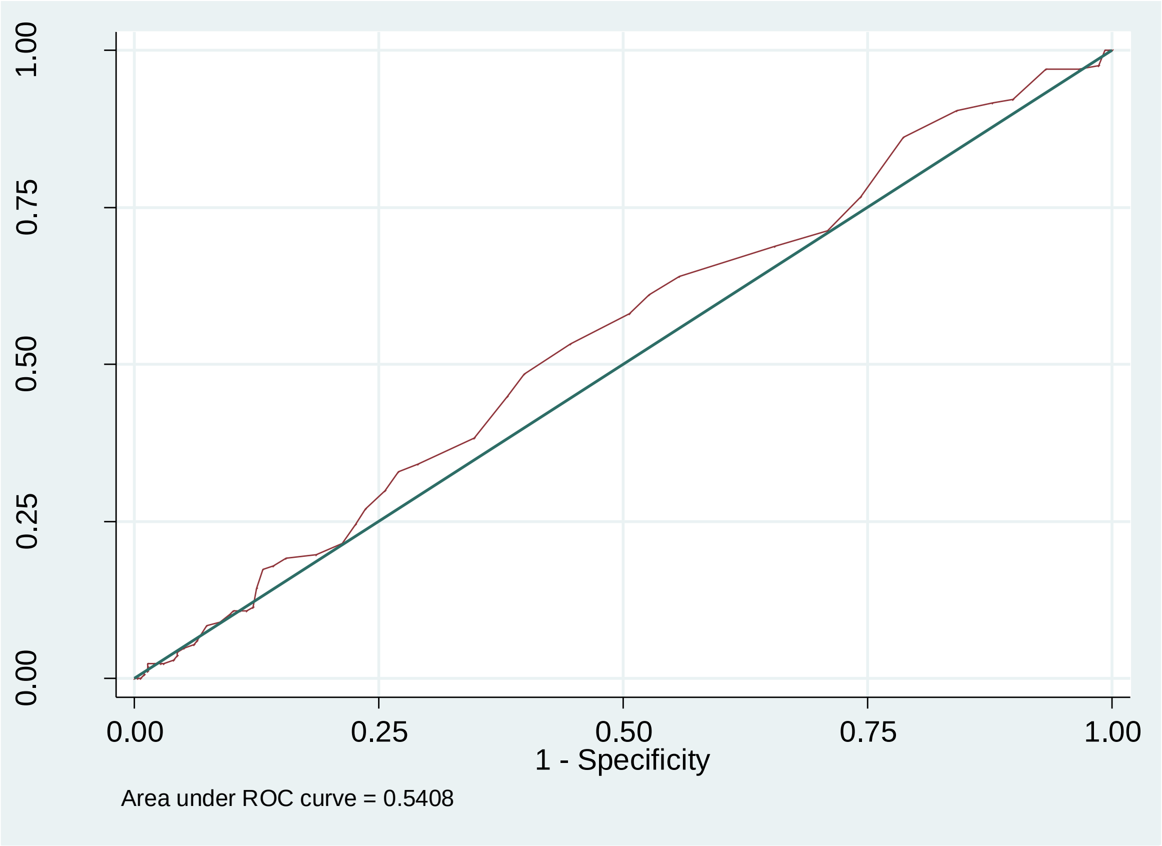

WHZ: weight-for-age z-score, MUAC: mid-upper arm circumference
